# An Analysis of Zonal Health Management Capacity and Health System Performance: Ethiopia Primary Healthcare Transformation Initiative

**DOI:** 10.34172/ijhpm.2022.6247

**Published:** 2022-02-22

**Authors:** Lingrui Liu, Mayur M. Desai, Tibebu Benyam, Netsanet Fetene, Temesgen Ayehu, Kidest Nadew, Erika Linnander

**Affiliations:** ^1^Global Health Leadership Initiative, Yale University, New Haven, CT, USA.; ^2^Department of Health Policy and Management, Yale School of Public Health, New Haven, CT, USA.; ^3^Department of Chronic Disease Epidemiology, Yale School of Public Health, New Haven, CT, USA.; ^4^Health Extension Program, Ethiopia Federal Ministry of Health, Addis Ababa, Ethiopia.

**Keywords:** Primary Healthcare, Sub-national Interventions, Health Management Capacity, System Performance, Ethiopia, Sub-Saharan Africa

## Abstract

**Background:** District management is emerging as a lynchpin for primary healthcare system performance. However, delivery of district-level interventions at scale is challenging, and overlooks the potential role of management at other subnational levels. From 2015-2019, Ethiopia’s Primary Healthcare Transformation Initiative (PTI), aimed to build a culture of performance management and accountability at the zonal level. This paper aims to evaluate the longitudinal change in management practice and performance in the 19 zones participating in PTI, which included 315 districts and 1617 health centers.

**Methods:** Using data from PTI intervention (2018 to 2019), we employed quantitative measures of management capacity at health center, district, and zonal levels, and quantified primary healthcare service performance using a summary score based on antenatal care coverage, contraception use, skilled birth attendance, infant immunization, and availability of essential medications. We used multiple generalized linear regression models accounting for clustering of health centers within zones to quantify (1) change in management and performance during the two-year intervention, (2) associations between the changes in management capacity at the zonal, district, and health facility level.

**Results:** Adherence to management standards at the zonal, district, and health facility level improved significantly over two years (37%, *P*<.001; 18%, *P*<.001; 18%, *P*<.001; respectively), as did the performance summary score (14%, *P*<.001). Adherence at the zonal level in year one was associated with district level adherence in year one (*P*=.04), and, over the two-year period (*P*=.002), and district management mediated the relationship between management practice at zonal and health center levels (*P*<.001).

**Conclusion:** Improvements in zonal-level management practice were associated with significant improvements in district-level management and performance in PTI sites. Investments in managerial practices at the zonal level may provide an immediate way to energize primary healthcare system performance at scale in low-income country settings.

## Background

**Key Messages**

** Implications for policy makers**

- Our study provides strong empirical evidence from a low-income country setting that can shape national strategies for performance improvement in primary healthcare systems.
- Managerial practices at the zonal level are associated with improved management practices at lower levels and improved primary healthcare system performance.
- Health management capacity and primary healthcare system performance at subnational levels (zonal, district, and health facility level) may be improved through health systems strengthening investments at the zonal level.

** Implications for the public**

To achieve universal health coverage targets and Sustainable Development Goals (SDGs), low-income countries are urged to pursue evidence-based approaches to improving primary healthcare system performance. Management capacity is a critical component of primary healthcare systems. However, existing evidence on the impact of investments in health management at the zonal level on concomitant changes in management practice and primary healthcare performance at the district and facility level is limited, especially in low-income country settings. Our study evaluates the impact of a longitudinal management strengthening intervention at the zonal level in Ethiopia. The results demonstrate that health management capacity and performance at sub-national levels can be measured using standardized tools, and that improvements in zonal level management is associated with improved management capacity and system performance at the district level.

The global health community has emphasized strengthening primary healthcare system performance as a central strategy for low-income countries to achieve universal health coverage targets and the Sustainable Development Goals (SDGs). Management, commonly defined as “the process of achieving predetermined objectives through human, financial, and technical resources”^1^ is foundational to health systems strengthening. Overcoming current threats to primary healthcare systems—declining external financing, uneven health workforces, and global shocks including the current coronavirus disease 2019 (COVID-19) pandemic—requires evidence-informed approaches to strengthening management capacity for a strong and resilient primary healthcare system.^1-5^

There is a growing body of evidence related to interventional approaches to strengthen management capacity at facility-level (eg, health center and hospital), many of which focus on quality improvement for a targeted set of health services.^6-9^ Management capacity at the district level is also emerging as a lynchpin for primary healthcare system performance based on cross-sectoral studies^6,10^ However, scaling-up of the proven facility- and district-level capacity building interventions, many of which include mentorship, coaching, or education as a key component, is costly and requires significant time to reach all geographies (for example, there are over 750 districts Ethiopia, compared to only 68 zones). Further, the focus on facility- and district-level interventions overlooks the potential role of management capacity at other subnational levels (zonal levels in Ethiopia; provinces or regions in other country contexts), levels that are especially important in the context of decentralization, which is becoming more common across countries.

 Therefore, we set out to evaluate the impact of a management strengthening intervention at the zonal level on concomitant changes in management practice and primary healthcare performance at lower levels of the health system (district health office and health center) using a longitudinal evaluation of practice and performance in sites associated with the Primary Healthcare Transformation Initiative (PTI) in Ethiopia. The results are expected to be useful to policy-makers developing national strategies for capacity development, implementers seeking to build management capacity in primary healthcare, and researchers seeking to reliably measure management capacity in low- and middle-income settings.

## Methods

###  Setting 

We conducted this study in Ethiopia, where 84% of the population lives in rural areas, 24% live below the poverty, and 44% are under the age of 15 years.^11^ Through rapid expansion of the primary healthcare system, Ethiopia has achieved remarkable progress in health outcomes, including significant reductions in preventable childhood and maternal mortality, and compelling decreases in communicable diseases. The government has articulated a vision for health system strengthening that prioritizes district-level capacity (*woreda transformation*).^12,13^ However, the performance of its primary healthcare system has been challenged as primary care professionals at the decentralized levels have struggled to absorb the new management roles and responsibilities required for the delivery of the proposed reforms.^14^

Ethiopia is divided into administrative regions, which are further divided into zones and then woredas (districts). The Zonal Health Department is responsible for primary healthcare services across 15-20 woredas (districts), each of which, on average, includes 4-5 health centers and the health extension program. Primary healthcare activities are organized and integrated at the district level, and PTI Phase I (2015-2017) effectively strengthened district management capacity.^6^ One level up, the zonal health department plays important roles in performance management, resource allocation, and cross-sectoral integration. Yet, the health management capacity at the zonal level was not well understood or developed.

###  Study Design

 We evaluated the impact of a management strengthening intervention in PTI Phase II (2018- 2019) at the zonal level (described below) on concomitant changes in management practice and primary healthcare system performance at lower levels of the health system (district health office and health center) using a longitudinal repeated measures design.

###  Intervention and Sample 

The Yale Global Health Leadership Initiative and Ethiopia Federal Ministry of Health (FMoH) established PTI with funding by the Bill & Melinda Gates Foundation. PTI aimed to build a culture of performance management and accountability at the zonal level, improving the effectiveness of districts in leading the ambitious set of primary healthcare reforms envisioned by the FMoH in its Health Sector Transformation Plan 2015-2020 and the accompanying Woreda Transformation Plan. In Phase I (2015-2017), PTI built management capacity in 36 woredas across four regions (Amhara, Oromia, SNNPR [Southern Nations, Nationalities, and People’s Region], and Tigray). A description of the project and the Phase I results have been published previously.^15^ In an attempt to influence change on a larger scale using similar resource investments (funding and staffing), PTI Phase II (2018-2019) focused on 17 zones (across Amhara, Oromia, and SNNPR) and 2 clusters (in Tigray, where the zonal structure is not used), including 315 woredas and 1617 health centers, serving a population of 47 million. Zones were selected for intervention through collaboration with regional health bureaus based on criteria including receptivity to the intervention, number of woredas, and size of population. The intervention included five levers of change: (1) Enhanced team and individual management and problem-solving capacity at the zonal level through a 6-month management certificate program; (2) Improved performance management practices through use of management standards and key performance indicators (KPIs); (3) Improved mechanisms for accountability, including use of managerial accountability measures and a Community Score Card; (4) Promotion of quarterly peer-review meetings at the zonal level for learning and support; and (5) Alignment of organizational structures with management functions.

###  Measures of Management Capacity at Health Center, District, and Zonal Levels

Leveraging the experiences in the development of the Woreda Management Standards (WMS) described below,^6^ PTI and FMoH collaborated to develop and test a set of corresponding Zonal Management Standards (ZMS) to measure and promote managerial capacity at zonal health departments. ZMS included 26 standards across five domains: governance and organizational capacity, health service delivery, community engagement, coordination with other sectors, and performance management (See Supplementary file 1, Tables S1 and S2 for details). For each zonal health department, adherence to ZMS was measured as the percentage of standards met. Adherence to ZMS was measured in Amhara, Oromia, SNNPR; Tigray does not have a zonal health department structure, thus the measure was not applicable.

As reported previously,^6,15^ we quantified management capacity at the woreda and health center level, using the WMS and the Ethiopia Health Center Reform Implementation Guidelines (EHCRIG). WMS consists of regionally- and nationally-endorsed 26 standards in 5 domains: governance and organizational capacity, service delivery, community engagement, collaboration with other sectors, and performance management.^6,15^ The percentages of standards met was assessed to measure the extent to which that each woreda health office be adhered to WMS and that each health center be adhered to EHCRIG, both overall and by domain, respectively. EHCRIG included 88 standards in 10 domains: leadership and governance, health post support, patient flow, medical records management, pharmacy services, laboratory services, infection prevention safety, medical equipment management, human resource management, and performance quality improvement^6,15^ (See Supplementary file 1, Tables S3 and S4 for details).

###  Measures of Primary Healthcare Service Performance 

As reported previously,^6^ PTI and FMoH developed and refined a KPI summary score composed of 5 measures: (1) contraceptive acceptance rate, ie, the number of women reporting use of modern contraception divided by the estimated number of women of childbearing age who are not pregnant in the health center catchment area; (2) antenatal care coverage, ie, the number of women having ≥4 antenatal care visits divided by the number of expected births in the health center catchment area; (3) skilled birth attendance rate, ie, the number of women who give birth in a health facility divided by the expected number of births in the health center catchment area; (4) the percentage of 1-year-old children who have received all recommended immunizations in the health center catchment area; and (5) essential drug availability, ie, the average percentage of 22 essential drugs to be found in stock per month at health centers.^6^ These 5 KPIs are a subset of the 18 KPIs prioritized by the FMoH and Regional Health Bureaus as part of the national Health Sector Transformation Plan. They were selected because they were most consistently reported with reliable data quality, indicated sufficient variation and room for improvement, and captured diverse aspects of system performance. For each health center, the five indicators, each normally distributed, were averaged to create a KPI summary score that could range from 0%-100%.

###  Data Collection 

As described previously,^6^ a PTI technical advisor for management systems in each site worked with their zonal health department counterparts to collect quarterly data on adherence to management standards (ZMS, WMS and EHCRIG), and performance on the KPIs after receiving training on the data collection tool and quality control activities, overseen by a PTI senior regional manager in each region. Health center adherence to EHCRIG standards was reported by facilities to district offices as part of Ethiopia’s health management information system and corroborated during routine supportive supervision visits by woreda health officers. Adherence to WMS was reported by the woreda health office to the zonal health office and corroborated via routine supportive supervision. Adherence to ZMS was measured by the PTI technical advisor for management systems through direct observation at the site. To evaluate the change in management capacity and performance over time, we consider quarterly data collected at two points in time: Health center level data (EHCRIG and KPIs) based on calendar year quarter 4 of 2017 and quarter 4 2018 and WMS and ZMS based on quarter 1 2018 and quarter 1 2019. Data were obtained from the woreda health offices and zonal health departments interviews with the key informants, review of relevant official documents and records, and direct observations.

###  Statistical Analysis

 We used standard descriptive statistics to characterize health center, woreda and zonal health department management capacity and performance at the three-time points relative to the two-year PTI intervention: baseline, the end of year one, and the end of year two. Specifically, we calculated the means and standard deviations of the ZMS (overall and by each of its five chapters), WMS, EHCRIG, and KPI summary scores.

 To examine the relationship between the changes of management capacity at zonal level and other levels (ie, district and health center levels), as well as the primary healthcare system performance, we used the generalized linear regression models. For each of the primary outcome variables (the absolute change of percentage points in WMS, EHCRIG, and KPI summary scores, respectively), we conducted two linear regression models, which examined the associations between the independent variable (the absolute change in ZMS score in year 1) and the outcome variable changes in year 1 and over the 2-year of the PTI intervention, respectively. We reported both the unadjusted and the adjusted associations of ZMS with the management capacity at levels of health centers and woreda health offices. The adjusted models were adjusted for the baseline ZMS and the baseline primary outcome variable. We used clustered standard errors to account for the non-independence of observations (health centers) within zones.

 In an additional closer analysis, we conducted linear regression models to examine the effect of the changes in specific aspects of zonal management practice in year 1 of the PTI intervention (independent variables) and the changes of woreda management capacity in year 1 and over the 2-year (outcome variables), respectively. We report both unadjusted and adjusted model findings. The adjusted models were adjusted for the baseline ZMS and the baseline primary outcome variables. Robust standard errors were adjusted for clustered design at the zonal level.

To determine whether woreda level management capacity was a potential mediator between the zonal level and health center level management capacity, we performed a mediation analysis.^16^ We assessed the total effect of zonal management capacity (ZMS, our independent variable) on the health center level management capacity (EHCRIG, dependent variable) through both direct and indirect effects. To do this, we conducted two additional linear regressions, one investigating the relationship of the independent variable (ZMS) with the mediator (WMS) and the other assessing the relationship of the mediator (WMS) with the dependent variable (EHCRIG) while controlling for the independent variable (ZMS).

There was no missing data for ZMS, and minimal missing data for WMS (1%) and EHCRIG (<3%). KPI indicator of essential drug availability is the least reported from the five KPI indicators: a missing of 29%; while the other four KPI indicators missing less than 6%. Records with missing data were dropped from the multivariate regression model analysis of the given outcome. Analyses were performed in Stata, version 15.1, and *P* < .05 was considered statistically significant.

## Results

 Our final sample included 1617 health centers in 315 woredas in 19 zones across four regions of Ethiopia. Regional distribution of health centers was 31% (n = 506) in Amhara; 40% (n = 644) in Oromia; 24% (n = 391) in SNNPR; 5% (n = 76) in Tigray. Of the woredas, 26% (n = 84) were in Amhara, 43% (n = 135) in Oromia, 25% (n = 79) in SNNPR, and 5% (n = 17) in Tigray.

Zonal, woreda, and health center-level management capacity (ZMS, WMS, and EHCRIG) improved across the overall sample and in each region (Table 1). The average ZMS score across 17 zones increased from 35.7% (standard deviation [SD] 12%) at baseline to 72.8% (SD 7%) at the end of the study period (*P* < .001). The average WMS score across 315 woredas increased from 42.1% (SD 17%) at the baseline to 60.5% (SD 15%) at the end of the study period (*P* < .001). The average EHCRIG score across 1617 health centers increased from 50.7% (SD 25%) at the baseline to 68.3% (SD 17%) at the end of the study period (*P* < .001). Similarly, the mean KPI summary score increased from 49% (SD 35%) at the baseline to 63.0% (SD 21%) at the end of one year (*P* < .001), and was sustained through the second year. Similar patterns of improvement in ZMS, WMS, EHCRIG, and KPI summary scores were found in all four regions.

**Table 1:** Management Capacity and Performance of PTI-Supported Health Centers, Woredas, and Zones in Ethiopia Over Time and by Region

**Outcome**	**Overall**			**Amhara**			**Oromia**			**SNNPR**			**Tigray**		
	**19 Zones, 315 Woredas, 1617 Health Centers**			**5 Zones, 84 Woredas, 506 Health Centers**			**7 Zones, 135 Woredas, 644 Health Centers**			**5 Zones, 79 Woredas, 391 Health Centers**			**2 Zones, 17 Woredas, 76 Health Centers**		
	**Baseline** ^a^	**End of ** **Year 1** ^b^	**End of ** **Year 2** ^c^	**Baseline**	**End of Year 1**	**End of Year 2**	**Baseline**	**End of Year 1**	**End of Year 2**	**Baseline**	**End of Year 1**	**End of Year 2**	**Baseline**	**End of Year 1**	**End of Year 2**
Management capacity at zonal health office: Mean (SD) ZMS score	35.7% (12)	57.3% (10)	72.8% (7)	38.7% (7)	63.3% (7)	73.5% (5)	28.9% (10)	54.1% (13)	74.8% (8)	43.2% (12)	54.9% (3)	68.51% (4)	NA	NA	NA
Chapter 1: Governance & organizational capacity	25.2% (8)	47.1% (14)	67.7% (8)	38.7% (7)	63.3% (7)	62.5% (4)	28.9% (10)	54.1% (13)	70.9% (9)	43.2% (12)	54.9% (3)	29.0% (7)	NA	NA	NA
Chapter 2: Service delivery	23.5% (13)	37.8% (14)	57.7% (17)	27.1% (7)	54.6% (2)	60.5% (13)	21.5% (8)	41.5% (14)	61.1% (16)	28.9% (7)	46.5% (17)	37.3% (17)	NA	NA	NA
Chapter 3: Community engagement	50.3% (19)	68.0% (15)	78.8% (10)	51.8% (13)	75.6% (13)	81.9% (8)	43.3% (18)	61.1% (17)	81.0% (8)	59.7% (22)	69.5% (8)	60.0% (22)	NA	NA	NA
Chapter 4: Coordination with other sectors	44.5% (23)	71.6% (15)	80.5% (14)	52.3% (20)	80.0% (15)	78.9% (9)	36.0% (21)	73.8% (19)	85.0% (17)	48.3% (25)	57.2% (16)	48.3% (25)	NA	NA	NA
Chapter 5: Performance management	35.3% (13)	62.2% (15)	79.2% (7)	42.7% (9)	67.6% (13)	83.6% (6)	25.7% (10)	55.0% (17)	76.1% (6)	41.6% (11)	66.9% (9)	41.6% (11)	NA	NA	NA
Management capacity at woreda health office: Mean (SD) WMS score	42.1% (17)	54.1% (16)	60.5% (15)	43.6% (18)	53.7% (14)	60.2% (16)	35.7% (12)	51.1% (17)	58.8% (15)	52.2% (16)	56.8% (17)	52.2% (16)	35.4% (4)	70.0% (8)	76.2% (8)
Management capacity at health center: Mean (SD) EHCRIG score	50.7% (25)	64.2% (19)	68.3% (17)	56.6% (21)	64.2% (17)	68.2% (15)	47.3% (25)	64.0% (19)	67.7% (15)	50.2% (29)	66.2% (21)	70.5% (19)	42.4% (20)	57.4% (24)	63.2% (24)
Health system performance: Mean (SD) KPI summary score	49.0% (35)	63.0% (21)	63.0% (20)	65.3% (27)	69.3% (17)	66.7% (18)	44.7% (34)	52% (22)	55.4% (19)	33.9% (40)	73.0% (19)	71.7% (20)	53.0% (27)	57.8% (18)	60.3% (16)

Abbreviations:PTI, Primary Healthcare Transformation Initiative; SNNPR, Southern Nations, Nationalities, and People’s Region; SD, Standard deviation; ZMS, Zonal Management Standards; KPI, key performance indicators; WMS, Woreda Management Standards; EHCRIG, Ethiopia Health Center Reform Implementation Guidelines; NA, not available. ^a^Baseline: the baseline assessment for management capacity at health center and health system performance were conducted in 2017 Q4, while the baseline assessment for leadership and management capacity at zonal level and woreda level health office were conducted in 2018 Q1. ^b^End of Year 1: the end of year 1 assessment for management capacity at health center and health system performance were conducted in 2018 Q4, while the end of year 1 assessment for leadership and management capacity at zonal level and woreda level health office were conducted in 2019 Q1. ^c^End of Year 2: the end of year 2 assessment for management capacity at health center and health system performance were conducted in 2019 Q3, while the end of year 2 assessment for leadership and management capacity at zonal level and woreda level health office were conducted in 2019 Q4. Note: The improvements in management practice at the zonal, district, and facility levels, and in KPIs from the baseline to the end of year 1, and from the baseline to the end of year 2, respectively, were statistically significant.

Multivariate regression models of the associations between the absolute change in ZMS, WMS, EHCRIG, and the KPI summary score show that improvement of zonal level management capacity in year-one was significantly associated with the improvement of woreda management capacity in year-one and across the two-year intervention period (Table 2). On the 0%-100% scale, for every 1-point increase in zonal management capacity in year-one, woreda management capacity increased 0.46 point in year one (95% confidence interval [CI]: 0.17 to 0.76, *P* = .004) and increased 0.41 point over the two-year intervention period (95% CI: 0.17 to 0.64, *P* = .002), respectively. The improvement in zonal level management capacity in year one was also positively associated with health center management capacity and healthcare system performance, but these relationships were not statistically significant.

**Table 2:** Results From Unadjusted and Adjusted Analyses of the Associations Between the Absolute Change of Zonal Management Capacity and the Absolute Change of the Primary Outcome Variables (WMS, EHCRIG, and KPI Summary Score)

	**Absolute Change in WMS (Percentage Points)**		**Absolute Change of EHCRIG (Percentage Points)**		**Absolute Change of KPI Summary Score (Percentage Points)**	
	**In Year 1 (1)**	**Over the 2-Year (2)**	**In Year 1 (3)**	**Over the 2-Year (4)**	**In Year 1 (5)**	**Over the 2-Year (6)**
**Absolute change in ZMS in year 1**						
Unadjusted (95% CI)	0.45 (0.22-0.69)^a^	0.54 (0.29-0.79)^a^	0.43 (0.07-0.8)^c^	0.45 (0.11- 0.79)^c^	0.01 (-0.47-0.49)	-0.19 (-0.42-0.04)
	n = 840	n = 805	n = 840	n = 805	n = 840	n = 805
Adjusted (95% CI)	0.46 (0.17-0.76)^b^	0.41 (0.17-0.64)^b^	0.09 (-0.15-0.32)	0.07 (-0.17- 0.30)	0.43 (-0.01-0.88)	0.01 (-0.30-0.31)
	n = 840	n = 805	n = 840	n = 805	n = 840	n = 805

Abbreviations:KPI, key performance indicators; WMS, Woreda Management Standards; EHCRIG, Ethiopia Health Center Reform Implementation Guidelines; ZMS, Zonal Management Standards; CI, confidential interval. Robust standard errors adjusted for clustered design at the zonal level. The adjusted model were adjusted for the baseline ZMS and the baseline primary outcome variable. ^a^ *P* < .001, ^b^*P* < .01, ^c^*P* < .05.

Upon a closer analysis (shown in Table 3), improvements in specific aspects of zonal management practice were significantly associated with the woreda management capacity. In the full adjusted model, zonal health department’s performance management capacity (ZMS domain 5) was predictive of improvement of WMS. On a 0%-100% scale, every 1-point increase in zonal performance management capacity in year-one, woreda management capacity increased 0.33 point in year-one (95% CI: 0.22 to 0.46, *P* < .001). The magnitude of the effect decreased slightly to 0.24 over the 2-year period but remained significant (95% CI: 0.04-0.45, *P* = .024). The improvements of two additional ZMS domains: service delivery (ZMS domain 2) and community engagement (ZMS domain 3) in year-one were also significant on the improvement of WMS over the two-year intervention period. Every 1-point increase in zonal service delivery and in zonal community engagement, woreda management capacity increased 0.16 point (95% CI: 0.01-0.32, *P* = .042) and 0.18 point (95% CI: 0.04- 0.32, *P* = .015) over the two-year intervention period, respectively.

**Table 3:** Results From Unadjusted and Adjusted Analyses of the Associations Between the Absolute Change of Individual Chapter Zonal Management Capacity and the Absolute Change of the Primary Variable Outcome

	**Absolute Change of WMS (Percentage Points) in Year 1**		**Absolute Change of WMS (Percentage Points) Over the 2-Year**	
**Absolute Change of ZMS in Year 1**	**Unadjusted (95% CI)** **n = 840**	**Adjusted (95% CI)** **n = 840**	**Unadjusted (95% CI)** **n = 805**	**Adjusted (95% CI)** **n = 805**
Chapter 1: Governance & organizational capacity	0.18 (-0.20-0.57)	0.18 (-0.14-0.49)	0.05 (-0.62-0.52)	0.04 (-0.27-0.29)
Chapter 2: Service delivery	0.16 (0.03-0.30)^c^	0.09 (-0.12-0.29)	0.28 (0.11-0.45)^b^	0.16 (0.01-0.32)^c^
Chapter 3: Community engagement	0.16 (-0.05-0.36)	0.08 (-0.16-0.31)	0.30 (0.13-0.46)^b^	0.18 (0.04-0.32)^c^
Chapter 4: Coordination with other sectors	0.07 (-0.10-0.25)	0.09 (-0.13-0.32)	0.10 (-0.09-0.28)	0.01 (-0.17-0.19)
Chapter 5: Performance management	0.36 (0.22- 0.51)^a^	0.33 (0.22-0.46)^a^	0.30 (0.05-0.55)^c^	0.24 (0.04-0.45)^c^
**Absolute Change of ZMS in Year 2**	**NA**		**Unadjusted (95% CI)** **n = 848**	**Adjusted (95% CI) ** **n = 848**
Chapter 1: Governance & organizational capacity	-		049 (0.16-0.81)^b^	0.20 (-0.22-0.62)
Chapter 2: Service delivery	-		0.19 (-0.08-0.46)	0.10 (-0.10-0.28)
Chapter 3: Community engagement	-		0.19 (0.05-0.34)^c^	0.16 (-0.10-0.42)
Chapter 4: Coordination with other sectors	-		0.10 (-0.11-0.28)	0.04 (-0.21-0.28)
Chapter 5: Performance management	-		0.48 (0.16-0.80)^b^	0.41 (0.10-0.72)^c^

Abbreviations:WMS, Woreda Management Standards; ZMS, Zonal Management Standards; CI, confidential interval; NA, not available. Robust standard errors adjusted for clustered design at the zonal level. The adjusted model were adjusted for the baseline ZMS and the baseline primary outcome variable. ^a^ *P* < .001, ^b^*P* < .01, ^c^*P* < .05.

The relationships of zonal- and woreda-management capacity improvements (in year-one: 95% CI: 0.36 to 0.55, *P* < .001; over the two-year: 95% CI: 0.44 to 0.64, *P* < .001) and the relationship of woreda- and health center- management capacity improvements (in year-one: 95% CI:0.03 to 0.20, *P* = .01; over the two-year: 95% CI: 0.09 to 0.24, *P* < .001) were significant (Table 4). The association between zonal- and health center- management capacity improvement was significantly mediated by woreda management capacity in year-one (95% CI: 0.24 to 0.50, *P* < .001) and over the two-year intervention period (95% CI: 0.24 to 0.49, *P* < .001). The proportion of the total effect of the ZMS improvement in year-one on EHCRIG improvement in year-one and over the two-year, that is mediated by the WMS improvement, is 0.12 and 0.19, respectively.

**Table 4:** Results of a Mediation Analysis Investigating Whether Woreda Level Management Capacity Mediates the Relationship Between Zonal Level and Health Center Level Management Capacity

**ZMS Absolute Change in Year 1 on EHCRIG Absolute Change in Year 1 (Total Effect)**	**ZMS Absolute Change in Year 1 on WMS Absolute Change in Year 1 (Total Effect)**	**WMS Absolute Change in Year 1 on EHCRIG Absolute Change in Year 1 Given ZMS Absolute Change in Year 1** ^c^	**ZMS Absolute Change in Year 1 on EHCRIG Absolute Change in Year 1 Given WMS Absolute Change in Year 1** ^d^	**Percent of Total Effect Mediated **	***P*** ** Value **
Estimate (95% CI)	Estimate (95% CI)	Estimate (95% CI)	Estimate (95% CI)	%	
0.42 (0.30- 0.55)^a^	0.45 (0.36- 0.55)^a^	0.11 (0.03- 0.20)^b^	0.37 (0.24- 0.50)^a^	0.12	<.001
**ZMS Absolute Change in Year 1 on EHCRIG Absolute Change Over the 2-Year Period (Total Effect)**	**ZMS Absolute Change in Year 1 on WMS Absolute Change Over the 2-Year Period (Total Effect)**	**WMS Absolute Change in Year 1 on EHCRIG Absolute Change Over the 2-Year Period Given ZMS Absolute Change Over the 2-Year Period** ^e^	**ZMS Absolute Change in Year 1 on EHCRIG Absolute Change Over the 2-Year Period Given WMS Absolute Change Over the 2-Year Period** ^f^	**Percent of Total Effect Mediated **	***P*** ** Value **
Estimate (95% CI)	Estimate (95% CI)	Estimate (95% CI)	Estimate (95% CI)	%	
0.45 (0.33- 0.57)^a^	0.54 (0.44- 0.64)^a^	0.16 (0.09- 0.24)^a^	0.36 (0.24- 0.49)^a^	0.19	<.001

Abbreviations:WMS, Woreda Management Standards; EHCRIG, Ethiopia Health Center Reform Implementation Guidelines; ZMS, Zonal Management Standards; CI, confidential interval. Interpretation: the proportion of the total effect of ZMS year 1 on EHCRIG year 1, and ZMS year 1 on EHCRIG over the 2-year, that is mediated by WMS is 0.12 and 0.19, respectively. ^a^ *P* < .001, ^b^*P* < .01. ^c^Effect of WMS absolute change in year 1 on EHCRIG absolute change in year 1 controlling for ZMS absolute change in year 1. ^d^Direct effect of ZMS absolute change in year 1 on EHCRIG absolute change in year 1 controlling for WMS absolute change in year 1. ^e^Effect of WMS absolute change in year 1 on EHCRIG absolute change over the 2-year period controlling for ZMS absolute change over the 2-year. ^f^Direct effect of ZMS absolute change in year 1 on EHCRIG absolute change over the 2-year period controlling for ZMS absolute change over the 2-year.

## Discussion

 We found that a two-year multifaceted intervention at the zonal level can significantly improve management capacity and performance at lower levels within the health system. Consistent with the intervention model, significantly greater improvement in management capacity was observed at the zonal level as compared to that at the woreda level and health center level, and woreda-level practice mediated the association between zonal- and health facility-level management. Investment in managerial practices at the zonal level provides a potential way to energize district-level managerial practices at scale, and specific aspects of zonal management practice may drive facility-level performance in low-income country settings.

This study builds upon PTI Phase 1 (2015-2017) by demonstrating significant associations between management practice at multiple levels in a much larger sample, and expanding our understanding of cross-sectional associations between management practice and performance established in prior studies.^6^ In the present study, the association between zonal management and KPI summary score was positive but not significant. This echoes previous findings^15^ that the improvements in managerial capacity may have a delayed impact on performance as they are swamped by the other factors in the system^17^ (such as reforms to national financing, supply chain, staffing, or infrastructure). Alternatively, management practice at the zonal level may be too far removed from service delivery to directly influence primary healthcare system performance, a hypothesis consistent with our findings of the important mediating influence of district management practice. Some stakeholders (eg, academic researchers, international/donors, policy-makers at various levels)^18,19^ have also hypothesized that improved managerial capacity may result in more accurate measurement and reporting of performance, which could result in an apparent decline in performance in the short term.

 Our study adds to the current body of the literature by providing longitudinal evidence that subnational healthcare management capacity (not only the health center level but also district and zonal level) can be improved in a relatively short time period. It is one of the first to demonstrate this impact in a large sample. It is also the first study to our knowledge that demonstrates the impact of investments in healthcare management capacity at zonal level on practices and performance at other sub-national levels (ie, woreda, and health center levels). Improving managerial practices at district and health center levels through a zonal-level intervention provides a way to energize primary healthcare system performance at scale in low-income country settings; notably, the Phase II approach drove change in 315 woredas using financial and human resources comparable to those required for 36 woredas in Phase I.

 The associations between improvements in specific chapters of the ZMS and improvements in ZMS highlights opportunities for further exploration. In Year 1, improvements in ZMS Chapter 5 (Performance Management) was most strongly associated with concurrent improvements in WMS, perhaps because of the speed with which performance management practices can drive results. Looking over a longer period, Year 1 improvements in ZMS Chapter 2 (Service Delivery) and Chapter 3 (Community Engagement) were also associated with two-year improvements in WMS. This may indicate that the effects of improvements in these domains are slower to come to fruition, but that they are ultimately powerful drivers of practice at lower levels of the system.

 Several limitations should be noted. First, PTI sites were selected in partnership with the government, not by random selection, to achieve diversity in geography and promote receptivity to the intervention. However, we observed relatively consistent results across regions and levels of the health system. Second, our observations were limited to a two-year period associated with a novel zonal level intervention; there may be a time lag in the associated change in performance, and we are unable to evaluate the sustainability of the gains achieved. Third, data quality can be a concern in low-resourced settings. In this study, however, the intervention itself had a focus on collection and use of data for improvement, and we used explicit protocols and provided rigorous training to staff to promote data quality. We believe that the remaining data quality issues were non-differential. Fourth, PTI was designed as a complex intervention to drive improvements in management practice, and the evaluation approach presented herein was intended to capture the impact of the PTI intervention as a whole, not to characterize the relative contribution of each of the five levers of change. Further research to identify the most salient aspects of the intervention approach could be a valuable contribution to the literature.

## Conclusion

The global health community has emphasized strengthening primary healthcare system performance as a central strategy for low-income countries to reach the health SDGs, with increasing acknowledgement of role of health management and leadership capacity.^4,20,21^ While many programs have focused on individual management competencies or behaviors,^22-25^ our study provides empirical evidence on how to systematically measure and improve subnational management systems to drive changes in primary healthcare system performance. To our knowledge, this is the first longitudinal study evaluating the impact of efforts to improve zonal-level management capacity in a low-income country. We demonstrate that zonal, district, and health facility level management capacity can be measured and improved in a relatively short period of time, with strengthening at the zonal level serving as an effective lever for change at the district and health center levels. The findings of this study will be useful to policy-makers developing national and subnational strategies for primary healthcare systems strengthening, professionals seeking evidence-based approaches to management and leadership capacity development in low- and middle-income settings, and researchers seeking to measure associations between management, leadership, and performance at multiple levels of the primary healthcare system.

## Ethical issues

 This study was exempted from human subjects review by the Human Subjects Committee of the authors institute, as it did not include individual-level protected health information.

## Competing interests

 Authors declare that they have no competing interests.

## Authors’ contributions

 Conceptualization: LL, MD, TB, NF, TA, KN, and EL. Data curation: LL, TB, and KN. Funding acquisition: EL. Methodology: LL, MD, and EL. Project administration: TB and KN. Supervision: MD and EL. Visualization: LL. Writing-original draft: LL. Writing-review: LL, MD, TB, NF, TA, KN, and EL.

## Funding

 Ethiopia’s Primary Healthcare Transformation Initiative (including this study) was funded by a grant to Yale University from the Bill & Melinda Gates Foundation.

## Supplementary files

Supplementary file 1 contains Tables S1-S4.
