# Supplementary material for: An Analysis of Zonal Health Management Capacity and Health System Performance: Ethiopia Primary Healthcare Transformation Initiative
Source: Int J Health Policy Manag. 2022 Feb 22;11(11):2610–7. doi: 10.34172/ijhpm.2022.6247 (PMC9818125; doi:10.34172/ijhpm.2022.6247)
Supplement: Supplementary file 1 — contains Tables S1-S4. [file ijhpm-11-2610-s001.pdf]

Article title: An Analysis of Zonal Health Management Capacity and Health System Performance: Ethiopia Primary Healthcare Transformation Initiative

Journal name: International Journal of Health Policy and Management (IJHPM)

Authors' information: Lingrui Liu[1,2]*, Mayur M. Desai[1,3], Tibebu Benyam[1], Netsanet Fetene[1], Temesgen Ayehu[4], Kidest Nadew[1,2], Erika Linnander[1,2]

[1]Global Health Leadership Initiative, Yale University, New Haven, CT, USA.

[2]Department of Health Policy and Management, Yale School of Public Health, New Haven, CT, USA.

[3]Department of Chronic Disease Epidemiology, Yale School of Public Health, New Haven, CT, USA.

[4]Health Extension Program, Ethiopia Federal Ministry of Health, Addis Ababa, Ethiopia

(*Corresponding author: Email: lingrui.liu@yale.edu)

Supplementary file 1

Table S1. Zonal Management Standards

**Chapter 1. Governance and organizational capacity**
1. The organizational structure of the Zonal Health Department reflects its core functions and comprising core processes and case teams responsible to execute the following core functions.
2. Zonal Health Department ensures governing boards of General Hospitals and Primary Health Care Facilities (HCs, PHs) are functional.
3. Zonal Health Department, Woreda Health Offices and Primary Health Care Facilities are led by qualified personnel
4. Zonal Health Department ensures General Hospitals, Woreda Health Offices and Primary Health Care Facilities are staffed.
5. Zonal Health Department ensure adequate finance allocation and provides financial oversight to General Hospitals, Woreda Health Offices and Primary Health Care Facilities (Budget vs. actual reports compiled by the Zonal Health Department)
6. Zonal Health Department provides oversight and facilitates procurement of goods and services by General Hospitals and Primary Health Care Facilities.
7. Zonal Health Department ensures that General Hospitals, Woreda Health Offices and Primary Health Care Facilities have basic infrastructure requirements: buildings, communications, electricity and water.
**Chapter 2. Health service delivery**
8. Zonal Health Department supports availability of secondary hospital services at General Hospitals and essential package of basic health care services at Primary Health Care Facilities (Review secondary health care services and essential package of basic health care services).
9. There is a referral and linkage system between General Hospitals and Primary Health Care Facilities in the Zone.
10. Zonal Health Department coordinates quarterly clinical audits in all Primary Health Care Facilities to ascertain adherence to clinical guidelines.
11. Zonal Health Department coordinates clinical mentoring between primary hospitals and health centers.
12. Monitor outbreak and public health emergencies (Surveillance report and Emergency response plan)
**Chapter 3. Community engagement**
13. Support woreda health offices in creating a verified model kebeles in the Zone.
14. Establish and maintain community feedback mechanisms (Town hall meeting minutes and Community score card report).

15. Zone health department starts and maintains Community Based Health Insurance (CBHI) scheme in all woredas.
16. Zonal Health Department coordinates community contribution and ownership on community based public health interventions.
**Chapter 4. Coordination with other sectors**
17. Coordinate execution of national and RHB initiatives by Woreda health offices and health facilities
18. Inter-sectoral coordination mechanisms established
19. Coordinate and align activities of development partners, and civil society organizations.
20. Private health facilities work in alignment with priorities of the woreda and operate within the national regulatory framework.
**Chapter 5. Performance management**
21. Zonal Health Department develops and reviews woreda based plan and targets.
22. System for performance review established and operational in General Hospitals, Woreda Health Offices and Primary Health Care Facilities.
23. Performance of General Hospitals, Woreda Health Office and Primary Health Care Facilities is monitored using evidence from; KPIs, EHRIG, EHCRIG and WMS.
24. Zonal Health Department establishes and implement bottom-up performance accountability mechanisms at zonal, woreda health and primary health facilities
25. Zonal Health Department compiles and disseminates national and regional policies, guidelines and manuals used as references for performance management.
26. Conduct supportive supervisions to General Hospitals, Woreda Health Offices and Primary Health Care Facilities

Appendix Table S2. Zonal Management Standards (detailed)

**Chapter 1. Governance and organizational capacity**
ZMS 1. The organizational structure of the Zonal Health Department reflects its core functions and comprising core processes and case teams responsible to execute the following core functions.
ZMS 1.1 Planning, monitoring and evaluation of health care services within the zone.
ZMS 1.2 Planning and coordinating supportive supervision, mentoring and technical support to General Hospitals, Woreda Health Offices and Primary Health Care Facilities
ZMS 1.3 Coordinate resource mobilization for General Hospitals, Woreda Health Offices and primary health care facilities.
ZMS 1.4 Ensuring community engagement and ownership
ZMS 1.5 Disease surveillance and coordinating and planning emergency response for public health emergencies.
ZMS 1.6 Conducting regulatory functions.
ZMS 1.7 Coordination with other sectors at the Zone level and linkage between RHB, General Hospitals and Woredas.
ZMS 1.8 Ensuring efficient utilization of financial resources by zonal health department
ZMS 1.9 Ensuring the proper human resources managements at zonal health departments
ZMS 1.10 Overseeing medical supplies and equipment to General Hospitals and Primary Health Care Facilities.
ZMS 1.11 Superintending the fulfilment of infrastructures to General Hospitals, Woreda Health Office and primary health care facilities
ZMS 2. Zonal Health Department ensures governing boards of General Hospitals and Primary Health Care Facilities (HCs, PHs) are functional.
ZMS 2.1 All governing boards of General Hospitals and primary health care facilities are functional. Zonal Health Department, presenting cases to the Zone administration/city mayor, ensures that corrective action is taken on governing boards that do not meet regularly.
ZMS 2.2 Minutes of governing board meetings include; review of action points from previous meeting, performance review using KPIs and way forward action points.

ZMS 2.3 Financial and programmatic performance targets are reviewed using woreda level Key Performance Indicators (KPIs), quarterly.
ZMS 2.4 Zone health department organizes governing board representatives from Zonal and Primary Hospitals (PHs) attend quarterly performance review meetings of the woredas.
ZMS 3. Zonal Health Department, Woreda Health Offices and Primary Health Care Facilities are led by qualified personnel
ZMS 3.1 Zonal Health Department head has the experience and educational qualification requirements (minimum of master degree in health related fields).
ZMS 3.2 Woreda Health Office heads have experience and educational qualification requirements (minimum degree in health and related fields).
ZMS 3.3 PHCU Directors have the experience and educational qualification requirements (minimum degree in health).
ZMS 3.4 CEOs of general and primary hospitals has the experience and educational qualification requirements (minimum of master degree in health and related fields).
ZMS 3.5 All General Hospitals, primary health care facilities and Zone health department managers have certificate-level, on job management, leadership, strategic problem-solving training.
ZMS 4. Zonal Health Department ensures General Hospitals, Woreda Health Offices and Primary Health Care Facilities are staffed.
ZMS 4.1 The Zonal Health Department review all requests from the Woreda health offices and hospitals each year for additional staff, and review these requests against the profile of existing staff based on regional staffing standards. Where appropriate, the ZHD submit special requests to the RHB.
ZMS 4.2 Zonal Health Department keeps updated record of Human Resource Information System(HRIS) of; Zonal Health Department, General Hospitals, Woreda Health Office and Primary Health Care Facilities (filled and vacant positions)
ZMS 4.3 The Zonal Health Department compile annual staffing pattern in each hospital, Woreda Health office and health facilities, make comparisons against the regional staffing standards to identify gaps, and submit bi-annual reports to the RHB on the staff currently employed.
ZMS 4.4 Zonal Health Department ensures that Zonal Health Department, General Hospitals, Woreda Health Offices and Primary Health Care Facilities are staffed with the required number and qualification of staff.
ZMS 4.5 Zonal Health Department ensures equitable allocation (professional mix and number) of health professionals among the General Hospitals and Primary Health Care Facilities in the woreda based on patient volume.
ZMS 4.6 Zonal Health Department recognizes and motivates high performing Zonal Health Department, General Hospitals and Woreda Health Office staff.
ZMS 5. Zonal Health Department ensure adequate finance allocation and provides financial oversight to General Hospitals, Woreda Health Offices and Primary Health Care Facilities (Budget vs. actual reports compiled by the Zonal Health Department)
ZMS 5.1 Zonal Health Department secures a minimum of 15% budget allocation to health from the total Zonal level administration expenditure.
ZMS 5.2 Zonal Health Department reviews income statements and balance sheets, and provides feedback to all General Hospitals, primary hospitals, Woreda Health Offices quarterly.
ZMS 5.3 Zonal Health Department monitors execution of internal financial audits of General Hospitals, woreda health office, Primary Health Care Facilities every six months.
ZMS 5.4 Zonal Health Department in coordination with zone finance plans and executes external financial audits for all General Hospitals, Woreda Health Offices and Primary Health Care Facilities annually.
ZMS 6. Zonal Health Department provides oversight and facilitates procurement of goods and services by General Hospitals and Primary Health Care Facilities.
ZMS 6.1 Zonal Health Department compiles procurement needs (goods and services) including essential drugs by General Hospitals and primary health care facilities under woredas.

ZMS 6.2 Zonal Health Department monitors that procurement are executed timely by General Hospitals, primary hospitals and health center and maintains checklist showing list of items requested vs. procured.
ZMS 6.3 Zonal Health Department has ensured implementation of LMIS (Logistics Management Information System) in General Hospitals and Primary Health Care Facilities.
ZMS 6.4 The Zonal Health Department oversees zero stock out rates across all essential drugs at Primary Health Care Facilities.
ZMS 6.4 The ZHD maintain records of all drugs and medical supplies it holds in stock, the amounts of each item requested by the health facilities and the amounts actually distributed, by date. A report of this data is submitted quarterly to the Zone's Management Committee for review.
ZMS 6.5 The ZHD coordinates quality assurance on primary health care facilities laboratory services.
ZMS 6.6 The ZHD maintains blood bank services to general hospital and primary health care facilities.
ZMS 6.7 The ZHD maintain an inventory of all items of equipment that require maintenance, vehicles and motorbikes by health facility and Woreda Health Office. This inventory is updated quarterly and included details of maintenance needs. The full inventory is submitted twice per year to the RHB.
ZMS 6.8 Coordinate quarterly equipment maintenance for zonal and primary health care facilities based on the identified needs.
ZMS 7. Zonal Health Department ensures that General Hospitals, Woreda Health Offices and Primary Health Care Facilities have basic infrastructure requirements: buildings, communications, electricity and water.
ZMS 7.1 Zonal Health Department keeps and update bi-annually a record of status of buildings, communications, electricity, and water availability for General Hospitals, Woreda Health Offices and primary health care facilities.
ZMS 7.2 Zonal Health Department bi-annually develops a plan of action in consultation with Woreda health offices, facility governing boards, Zone and woreda Administration and other relevant stakeholders to fill identified infrastructure gaps in building, communications, electricity, and water.
**Chapter 2. Health service delivery**
ZMS 8. Zonal Health Department supports availability of secondary hospital services at General Hospitals and essential package of basic health care services at Primary Health Care Facilities (Review secondary health care services and essential package of basic health care services).
ZMS 8.1 Zonal Health Department maintains an updated record of secondary health care services provided at General Hospitals and essential package of basic health care services available in each Primary Health Care Facilities.
ZMS 8.2 Zonal Health Department ensures secondary health care services are provided at General Hospitals and essential package of basic health care services are available in each Primary Health Care Facilities.
ZMS 9. There is a referral and linkage system between General Hospitals and Primary Health Care Facilities in the Zone.
ZMS 9.1 Zonal health department ensures all general and Primary Health Care Facilities use standard referral and feedback protocol including standard referral and feedback forms and registers.
ZMS 9.2 Zonal Health Department organizes quarterly referral feedback meeting between general hospitals and Primary Health Care Facilities where data on referral, feedback, and unnecessary referrals are reviewed.
ZMS 9.3 Ambulance administration policy is in place and operational in General hospitals and all primary health care facilities.
ZMS 10. Zonal Health Department coordinates quarterly clinical audits in all Primary Health Care Facilities to ascertain adherence to clinical guidelines.
ZMS 10.1 Zonal Health Department identifies clinical audit areas for respective secondary and primary health care facility in collaboration with the Health Care Facilities.
ZMS 10.2 Zonal health department prepares, adapts and updates clinical audit tools in identified clinical audit areas.
ZMS 10.3 Zonal Health Department monitors General hospitals and Primary Health Care Facilities conduct clinical audits on the areas identified quarterly.

ZMS 10.4 Zonal Health Department ensures implementation of quality improvement activities in all Primary Health Care facilities based on clinical audit findings.
ZMS 11. Zonal Health Department coordinates clinical mentoring between primary hospitals and health centers.
ZMS 11.1 Zonal Health Department working with the CEO of General hospitals, Woreda Health Offices and Primary Hospitals develops schedule of clinical mentoring sessions between Zonal, primary hospital and all health centers. (Clinical mentoring may include one-on-one case management and observation, chart reviews, attachments, and didactic sessions).
ZMS 11.2 Zonal Health Department organizes knowledge and skills assessments of mentees semi-annually in collaboration with General Hospitals, Woreda Health Offices, and primary hospital to monitor the outcome of mentoring.
ZMS 11.3 Zonal Health Department organizes trainings or mentoring sessions in collaboration with the Zonal and primary hospital based on knowledge and skills gap identified.
ZMS 12. Monitor outbreak and public health emergencies (Surveillance report and Emergency response plan)
ZMS 12.1 Integrated Diseases Surveillance and Response (IDSR) is in place at Zonal health department, Woreda Health Offices and health facilities.
ZSM 12.2 Resources and systems are prepared to respond to public health emergencies.
**Chapter 3. Community engagement**
ZMS 13. Support woreda health offices in creating a verified model kebeles in the Zone.
ZMS 13.1 Zonal Health Department ensures all woreda health offices compiled and analyses health extension package performance of all kebeles and categorizes them into; high, middle and low performers.
ZMS 13.2 Zone health department supports woredas in organizing annual mobilization (ignition) meeting with the community focusing on creation of model kebeles.
ZMS 13.3 Zonal Health Department monitors performance in creating model kebele initiative in all woredas quarterly.
ZMS 14. Establish and maintain community feedback mechanisms (Town hall meeting minutes and Community score card report).
ZMS 14.1 A system of community score card is established and maintained at General Hospitals and Primary Health Care Facilities quarterly.
ZMS 14.2 Zonal Health Department working closely with the Zone administration, general and primary Hospital, organizes quarterly community town hall meetings where community provides feedback on quality and access to services for the hospitals.
ZMS 14.3 Zonal Health Department in consultation with General Hospitals and Primary Health Care Facilities, coordinates the implementation of activities responding to feedback from the community quarterly.
ZMS 15. Zone health department starts and maintains Community Based Health Insurance (CBHI) scheme in all woredas.
ZMS 15.1 Zonal health department ensures all woredas meets and maintains minimum community enrollment and renewal to start and continue CBHI.
ZMS 15.2 Zonal health department monitors CBHI performance of the woredas and provides feedback.
ZMS 15.3 Zonal Health Department in collaboration with zonal administration organizes quarterly meetings with CBHI agency, Woreda Health Offices, General Hospitals and PHC facilities to review performance progress on CBHI.
ZMS 16. Zonal Health Department coordinates community contribution and ownership on community based public health interventions.
ZMS 16.1 Zonal Health Department identified and prepared plan on health projects which can be implemented in monetary and in-kind support from the community.
ZMS 16.2 Zonal Health Department coordinates monetary and in-kind community contributions in infrastructure projects such as building health posts, HEW residences, community latrines, maternity waiting areas, and other projects.

ZMS 16.3 Zonal Health Department oversees and coordinates all monetary and in-kind contributions of the community and reports back to community on achievements.
**Chapter 4. Coordination with other sectors**
ZMS 17. Coordinate execution of national and RHB initiatives by Woreda health offices and health facilities
ZMS 17.1 Zonal Health Department regularly keep updates of national and regional health bureau initiatives, guidance and link to the woreda health office and health care facilities.
ZMS 17.2 Zonal health department monitor implementation progress of national and regional initiatives at woreda and health care facilities.
ZMS 17.3 Provide feedback to woredas and health care facilities on their level of implementing national and RHB initiatives.
ZMS 18. Inter-sectoral coordination mechanisms established
ZMS 18.1 Zonal Health Department ensures integration of health sector related activities with other sectors' plans such as education, agriculture, infrastructure, electricity, water, finance, civil service, and others at Zonal level.
ZMS 18.2 Zonal Health Department implemented joint planned activities with other sectors.
ZMS 18.3 Zonal level inter-sectorial steering committees monitor jointly planned activities and makes decisions to address potential bottlenecks quarterly.
ZMS 19. Coordinate and align activities of development partners, and civil society organizations.
ZMS 19.1 Zonal Health Department maintains updated mapping of development partners, and civil society organizations working in the health sector.
ZMS 19.2 Zonal Health Department works with the Zonal administration to organize joint planning and review meetings with development partners, and civil society organizations working in the health sector quarterly.
ZMS 20. Private health facilities work in alignment with priorities of the woreda and operate within the national regulatory framework.
ZMS 20.1 Zonal Health Department has included all private facilities related with the health sector in the zone such as; private health facilities, food and drink provides, schools, industrial sites, etc. in its regulatory plan.
ZMS 20.2 Zonal Health Department organizes periodic inspections in private facilities based on FMHACA regulatory standards and set up a system for follow-up.
ZMS 20.3 Zonal Health Department supports woreda health office in organizing joint consultations with private facilities semi-annually.
**Chapter 5. Performance management**
ZMS 21. Zonal Health Department develops and reviews woreda based plan and targets.
ZMS 21.1 Zonal Health Department develops 5-year strategic plan for the zone.
ZMS 21.2 Zonal Health Department supports all woredas develop woreda based annual plan and targets aligned with the strategic plan and allocates targets to Zonal and Primary Health Care Facilities.
ZMS 21.3 Zonal Health Department organizes quarterly EHIAQ (Ethiopian health institutions Alliance for Quality) review meetings with the participation of General Hospitals, primary hospitals and Woreda Health Offices to review implementation of activities and provide feedback.
ZMS 21.4 Zonal Health Department supports all woredas and health facilities conduct an effective quarter review meetings using Ethiopia Primary Health Care Alliance for Quality (EPAQ) approaches
ZMS 21.5 Zonal Health Department facilitate experience sharing visits bi-annually among woredas and health facilities based on level of performance using EPAQ approaches.
ZMS 21.6 Zonal Health department conducts recognition for high performing woredas and health facilities bi-annually using EPAQ approaches.

ZMS 22. System for performance review established and operational in General Hospitals, Woreda Health Offices and Primary Health Care Facilities.
ZMS 22.1 Zonal Health Department, General Hospitals, Woreda Health Offices and all Primary Health Care Facilities conduct quarterly performance reviews based in balanced score card (staff BSC) and semi-annually 360 performance evaluations (case team).
ZMS 22.2 Zonal Health Department monitors implementation of result oriented performance review in General Hospitals, Woreda Health Office and Primary Health Care Facilities semi-annually and ensure performance review results are used for human resource and management decisions.
ZMS 23. Performance of General Hospitals, Woreda Health Office and Primary Health Care Facilities is monitored using evidence from; KPIs, EHRIG, EHCRIG and WMS.
ZMS 23.1 Zonal Health Department, General Hospitals, Woreda heath office and primary health facility managers and boards use KPIs to monitor performance and take corrective action on a quarterly basis.
ZMS 23.2 Zonal Health Department verifies Woreda management standard (WMS) assessment of woredas and EHSTG assessment of General Hospitals quarterly.
ZMS 23.3 Zonal health department ensures woreda health offices verify EHCRIG assessments of health centers and EHSTG assessment of primary hospitals.
ZMS 23.4 Zonal Health Department monitors HMIS data quality including; timeless, accuracy, completeness, reliability of data from woreda health offices and Primary Health Care Facilities and provide feedback based on Routine Data Quality Assessment (RDQA) ZHD conducted quarterly.
ZMS 24. Zonal Health Department establishes and implement bottom-up performance accountability mechanisms at zonal, woreda health and primary health facilities
ZMS 24.1 The zonal health department, woreda health offices and primary health facilities initiated use of bottom-up performance accountability mechanisms.
ZMS 24.2 The zonal health department and woreda health offices utilizes the feedback provided through bottom-up accountability mechanisms for its performance improvement.
ZMS 25. Zonal Health Department compiles and disseminates national and regional policies, guidelines and manuals used as references for performance management.
ZMS 25.1 Zonal Health Department compiles and disseminate national and regional policies, guidelines, and manuals to General Hospitals, Woreda Health Offices and all Primary Health Care Facilities.
ZMS 25.2 Zonal Health Department monitors availability of a list of policies, guidelines and manuals at General Hospitals, Woreda Health Office and Primary Health Care Facilities quarterly.
ZMS 26. Conduct supportive supervisions to General Hospitals, Woreda Health Offices and Primary Health Care Facilities
ZMS 26.1 Zonal Health Department uses standardized integrated supportive supervision checklist customized to each level of General Hospitals, Woreda Health Offices and Primary Health Care Facilities and community level.
ZMS 26.2 Zonal Health Department conducts integrated supportive supervision quarterly, jointly with relevant stakeholders, and written feedback is given to all General Hospitals, Woreda Health Office and Primary Health Care Facilities.
ZMS 26.3 Zonal Health Department puts in place a system for tracking action on feedback provided through supportive supervisions.

Table S3. Woreda Management Standards

**Chapter 1: Governance and Organizational Capacity**
1. The organizational structure of the Woreda Health Office reflects its core functions. The organizational structure of the Woreda Health Office has core processes and case teams responsible to execute the following core functions.
2. Woreda Health Office ensures governing boards of Primary Health Care Facilities (HCs, PHs) are functional.
3. Coordination and communication among governing boards of Primary Health Care Facilities.
4. Woreda Health Office and Primary Health Care Facilities is led by qualified personnel
5. Woreda Health Office ensures Primary Health Care Facilities are staffed.
6. Woreda Health Office ensure adequate finance allocation and provides financial oversight to Primary Health Care Facilities (Budget vs actual reports compiled by the Woreda Health Office)
7. Woreda Health Office provides oversight and facilitates procurement of goods and services by Primary Health Care Facilities.
8. Woreda Health Office ensures that Primary Health Care Facilities have basic infrastructure requirements: buildings, communications, electricity and water.
**Chapter 2: Service Delivery**
9. Woreda Health Office ensures availability of essential package of basic health care services at Primary Health Care Facilities.
10. There is a referral and linkage system between Primary Health Care Facilities in the Woreda.
11. Woreda Health Office coordinates quarterly clinical audits in all Primary Health Care Facilities to ascertain adherence to clinical guidelines, SOPs.
12. Woreda Health Office coordinates clinical mentoring between primary hospital and health centers.
13. Monitor outbreak and public health emergencies(Surveillance report and Emergency response plan)
**Chapter 3: Community Engagement**
14. Community are organized in 1-5 networks and developments teams(Health Development group command post reports).
15. All kebeles in the Woreda are verified as model in health service delivery.
16. Establish and maintain community feedback mechanisms (Town hall meeting minutes and Community score card report).
17. Woreda starts and maintains Community Based Health Insurance (CBHI) scheme.
18. Woreda Health Office coordinates community contribution and ownership on community based public health interventions.
**Chapter 4: Coordination with other key sectors in the Woreda**
19. Inter-sectoral coordination mechanisms established.
20. Coordinate and align activities of development partners, and civil society organizations.
21. Private health facilities work in alignment with priorities of the Woreda and operate within the national regulatory framework.
**Chapter 5: Performance Management**
22. Woreda Health Office develops Woreda based plan and targets.
23. System for performance review established and operational in Primary Health Care Facilities.
24. Performance of Primary Health Care Facilities is monitored using evidence fromKPIs, EHRIG, EHCRIG.
25. Woreda Health Office compiles and disseminates national and regional policies, guidelines and manuals used as references for performance management.
26. Supportive supervisions to Primary Health Care Facilities (Supportive supervision report)

Table S4. Ethiopia Health Center Reform Implementation Guidelines (EHCRIG) domains and standards

**Domain 1. HEALTH CENTER LEADERSHIP AND GOVERNANCE**	
1.	The Health Center Governing Board is established
2.	The Health Center Governing Board conducts regular meeting at least once in three months
3.	The Body approves annual and strategic plans for the health center to achieve its goal of improving its community's health and welfare and evaluate its achievement versus plan every quarter.
4.	The health center director is assigned by the city administration or woreda health office. The health center director organize and lead the heath center management committee comprised of different case teams.
5.	The health center director is evaluated quarterly, consistent with operational and strategic plans as established by the Body and the manager collectively.
6.	Health center director plan versus achievement report is monthly submitted to the wored health office
7.	Free and fee for health services that clients pay at reception and cashier are posted at health notice board in clear and visible manner
8.	The health center has signed memorandum of understanding agreement with entities that it provides free or credit health care services
9.	The health center finance officer presents the detailed status of health center financial reports to the health center management committee
10.	The health center has procurement plan approved by the health center governing body
11.	Health center financial manual is approved by the health center governing board and bears the seal of the of health center on it
12.	The health center finance is at least annually audited by the woreda finance office
**Domain 2. HEALTH POST SUPPORT**	
13.	The health center has established the necessary structural arrangement to implement linkage with health posts including assigning personal to manage and follow the system.
14.	A one to five linkage is established in the catchment community and the functionality is assured
15.	The health center creates linkage with 5 health posts under its custody and ensures its functionality
16.	The health center prepares core plans as a PHCU for the health posts under its custody and engage the community and relevant stallholders
17.	A detailed time table and plan is developed for each professional in the health center to support health extension workers at each Development Group.
18.	The health center collect and analyze weekly performance reports from health posts and organize a regular review meetings to follow the overall progress in major community activities, to identify best practices and to share it among health posts.
19.	The health center sends a timely and appropriate feedback to the health posts and follows the implementation of the feedback during regular support.
20.	The health center organizes an integrated and a regular supportive supervision to identify major gaps and to support accordingly.
21.	The health center allocates appropriate supplies to health posts and follows their appropriate use.
**Domain 3. PATIENT FLOW**	
22.	Procedures are established to ensure efficient patient flow; such procedures are specific to emergency, outpatient and delivery services that seek to reduce patient crowding.
23.	All health center staff are aware on the procedures guidelines and implement accordingly
24.	The Health center has a Triage, staffed with appropriately trained personnel and equipped with necessary equipment and supplies.

25.	Outpatient/client appointment systems are in place for all disciplines provided by the health center.
26.	The Health center has a Liaison and Referral Service guideline that the health facility staff understand and implement
27.	There are sign posts at the compound of the health that directs clients/patients to the desired health service units
28.	The health center has established maternity waiting room fulfilled with essential utilities such as latrine, bathroom, electric power and water
**Domain 4. MEDICAL RECORDS MANAGMENT**	
29.	The Health Center utilizes a single, unified registration system for all patients/clients, including out-patients and emergency admissions (if relevant, inpatient) or specialty clinics and also utilizes a Master Patient Index with a single, unique Medical Record Number for each patient.
30.	The Health Center utilizes a paper-based or computer-based system to track where the medical record is located at all times and also uses a standardized and uniform set of forms prepared by the FMOH or RHB to document a complete medical record for patient's/client's care.
31.	The Health Center has medical records management guidelines for proper handling and confidentiality of medical records
32.	The Health Center has orientation and training programs for all medical records personnel to ensure awareness and competency in medical record management procedures
**Domain 5. PHARMACY SERVICES**	
33.	The Health Center has a Drug and Therapeutics Committee (DTC) which implements various measures designed to promote the rational, safe and cost-effective use of medicines.
34.	The Health Center has a separate pharmacy department comprising dispensaries and medical store directed by a registered Pharmacist and Pharmacist/Pharmacy technician respectively.
35.	The Health Center has a health facility specific List of Medicines classified by VEN that contains all Drugs, Medical Supplies, consumable-Medical equipment's and Reagents. The List shall be reviewed and updated annually.
36.	The health center has an effective supply chain system for pharmaceuticals, medical equipment and supplies
37.	The health center provides clear and correct prescribed drug information to patients and keep the proper record of the service
38.	The Health Center provides access to drug information to both health care providers and patients in order to optimize drug use.
39.	The Health Center has policies and procedures for identifying and managing drug use problems, including: Identifying and reporting adverse drug reactions, and prescription monitoring
40.	The Health Center has a supply and inventory management system for drugs, medical supplies and consumable equipment approved by the DTC
41.	The Health Center conducts a physical inventory of all pharmaceuticals in the store and each dispensing unit at a minimum once a year.
42.	The Health Center ensures proper and safe disposal of pharmaceutical wastes and expired drugs in line with national guidance.
43.	The health centers pharmacy assists and monitors pharmaceutical management activities at the health posts.
44.	All Units of the pharmacy service have adequate personnel, equipment, premises and facilities required to store drugs, medical supplies and equipment and carry out dispensing, and counselling services.
45.	The health center conducts internal audit twice and external audit once every year on all pharmaceuticals, equipment and supplies
**Domain 6. LABORATORY SERVICES**	

46.	Current list of laboratory tests provided by the facility with the price of each test is accessible to all clinical staff and patients.
47.	Does the Laboratory management meet the needs and requirements of customers making the laboratory results are discussed up on and interpreted correctly
48.	Does the Laboratory have adequate number of staff, necessary space, and materials for work?
49.	The laboratory has a logistic management system to monitor the procurement and use of laboratory materials that prevents unnecessary storage or shortage
50.	The laboratory has standard operating procedures(Rejection, transport, retention and disposal) and follows it properly
51.	The laboratory work environment is organized and clean at all times that specimen handling mechanisms ensures safety for the service providers and users
52.	The laboratory have a health and safety manual with procedures that include different types of actions(handling fire and chemical hazard etc.)
53.	The laboratory management prepares an established policy for data and information management on data safety, confidentiality, data storing period and disposal mechanisms
54.	Does the laboratory have and implements a quality assurance policy that covers all aspects of laboratory functions
**Domain 7. INFECTION PREVENTION SAFETY**	
55.	Infection prevention and patient safety committee is established
56.	Infection prevention and patient safety committee implementation guideline is prepared
57.	Infection prevention and patient safety committee has prepared action plan
58.	Infection prevention and patient safety committee plan vs performance report is reviewed by the health center management body
59.	Major communicable diseases at the health centers are identified
60.	Standard practices to prevent, control and reduce risk of HCAIs are in place and transmission based precautions (TBP) are adequately addressed
61.	Health center infection prevention activity monitoring and implementation is based on routes of infection transmission
62.	The Health center ensures that equipment, supplies and facilities/infrastructure necessary for infection prevention are available.
63.	In order to implement Infection prevention standard and patient safety activities, essential antiseptic and disinfectants are fulfilled in sufficient and appropriate composition
64.	All health center staff are trained using standard infection prevention training materials.
65.	The Health center provides health education to patients, caregivers and visitors, as appropriate on infection prevention practices
66.	Infection prevention and patient safety activities at the health center are also harmonized with the satellite health post and health development groups activity at community level
**Domain 8. MEDICAL EQUIPMENT MANAGMENT**	
67.	The health center conducts annual inventor and has a paper-based or computer-based inventory management system that tracks all equipment included in the equipment management program.
68.	Health center assigned head of medical equipment technicians accountable to the head of the health center
69.	All new equipment undergoes acceptance testing prior to its initial use to ensure the equipment is in good operating condition. Equipment is installed and commissioned in accordance with the manufacturer's specifications.

70.	All equipment users are appropriately trained on the operation and maintenance of medical equipment with standard operating procedures readily available to the user. All equipment users are trained on emergency and fire accident prevention and control techniques
71.	The health center owns a building plan that is registered by its name, fenced compound, well functioning waste disposal system, toilet and clean and safe and green recreational/garden areas
72.	There is medical equipment and facility engineering administration policy manual /guidance at health center indicating the health center receives Medical equipment maintenance technical support from selected joint coordination bodies such as hospital, regional health bureau or there outsourced institution based on contractual or MOU agreement
73.	For health center medical engineering administration there is a medical equipment maintenance request, opening, closing and reporting formats readily available
74.	The health center established system on use of facility cars with formal entry and exit control at the facility gates
75.	For health center essential health service care, there is 24/7 water and electric power supply or its alternatives
76.	Health center has an advance emergency preparedness and response plan on fire and emergency conditions
**Domain 9. HUMAN RESOURCE MANAGMENT**	
77.	Personnel are assigned to provide Human Resource Management services.
78.	Each employee has a personnel file that is maintained by the HRM case team or case worker.
79.	A Human Resource Development plan has been developed
80.	Job descriptions have been adopted and documented for each position at the health center.
81.	Performance evaluation has been made every 6 month for each each employee.
82.	Employee benefits and motivation package developed and implemented.
83.	Standardized uniforms and badges are worn by staff.
**Domain 10. PERFORMANCE QUALITY IMPROVEMENT**	
84.	The health center should establish a structure (multidisciplinary team) for performance monitoring and quality improvement
85.	The health center needs to prepare an annual and quarterly plan
86.	The health center needs to implement quality improvement cycle for selected priority problems
87.	The health center needs to collect, analyze and use for quality improvement purposes and report reportable indicators to the respective body as per the HMIS standard
88.	Client satisfaction survey and other quality improvement assessments are conducted, analyzed and applied for quality improvement purposes
